# Analysis of the Properties of Fractional Heat Conduction in Porous Electrodes of Lithium-Ion Batteries

**DOI:** 10.3390/e23020195

**Published:** 2021-02-05

**Authors:** Xin Lu, Hui Li, Ning Chen

**Affiliations:** 1 Department of Mechanical and Electronic Engineering, Nanjing Forestry University, Nanjing 210037, China; luxin2017@foxmail.com; 2 SVOLT Energy Technology Co., Ltd., Wuxi 214000, China; lihui@svolt.cn

**Keywords:** lithium-ion battery, heat conduction, fractional calculus, temperature field

## Abstract

Research on the heat transfer characteristics of lithium-ion batteries is of great significance to the thermal management system of electric vehicles. The electrodes of lithium-ion batteries are composed of porous materials, and thus the heat conduction of the battery is not a standard form of diffusion. The traditional heat conduction model is not suitable for lithium-ion batteries. In this paper, a fractional heat conduction model is used to study the heat transfer properties of lithium-ion batteries. Firstly, the heat conduction model of the battery is established based on the fractional calculus theory. Then, the temperature characteristic test was carried out to collect the temperature of the battery in various operating environments. Finally, the temperature calculated by the fractional heat conduction model was compared with the measured temperature. The results show that the accuracy of fractional heat conduction model is higher than that of traditional heat conduction model. The fractional heat conduction model can well simulate the transient temperature field of the battery. The fractional heat conduction model can be used to monitor the temperature of the battery, so as to ensure the safety and stability of the battery performance.

## 1. Introduction

New energy vehicles have attracted more and more attention due to their significant advantages in energy conservation and emission reduction. As a key component of electric vehicles, the power performance lithium-ion batteries directly affects the promotion of electric vehicles [1]. Temperature has an important influence on the overall performance of lithium-ion batteries [2,3]. Lithium-ion batteries generate a lot of heat during the charge–discharge process, causing the battery temperature to rise. The uneven temperature distribution will affect the performance and cycle life of the battery, and in severe cases it will affect the safety and reliability of the battery [4,5]. Therefore, it is of great significance to study the heat transfer characteristics of lithium-ion battery during operation.

The mathematical model of thermal effect is a basic tool to study the heat transfer properties of lithium-ion batteries. The thermal effect model can describe the thermal behavior of the battery under different conditions, thereby simulating the temperature field of the battery. According to the principle of heat generation, the thermal model of lithium-ion battery can be divided into the electric–thermal coupling model, the electrochemical–thermal coupling model, and the thermal abuse model. The electrochemical–thermal coupling model simulates the heat generation of the battery based on the electrochemical reaction mechanism [6,7,8]. The electrochemical–thermal coupling model generally assumes that the current density distribution of the cell is uniform. In the temperature simulation of small batteries, the electrochemical–thermal coupling model can ensure the accuracy of the model. The electric–thermal coupling model studies the temperature distribution of the cell according to the distribution of the current density. The electric–thermal coupling model can be used to guide the improvement of the shape, tabs, and current collectors of the battery [9,10,11,12,13]. The thermal abuse model combines the traditional thermal model with the possible thermal reaction in the battery. The thermal abuse model can predict the state variation of the battery during the thermal runaway.

Currently, the mainstream lithium-ion battery is composed of porous electrodes and porous separators. The porous electrode of lithium-ion battery has the discontinuity and self-similarity in the microscopic view [14,15]. The heat conduction in porous electrodes is not a standard thermal diffusion model. The traditional heat conduction model is not suitable for describing the non-standard heat conduction phenomenon in porous electrodes [16,17,18]. Dynamic systems in porous materials, discontinuous materials, polymers, and composite materials usually have some nonlinear characteristics, such as the dependence on historical states, the discontinuities, the fractal characteristics, and the chaotic characteristics [19,20]. In recent years, fractional calculus theory has become a hot topic in the study of nonlinear problems. Fractional calculus is widely used in the fields of oscillation, diffusion, elasticity, non-Newtonian fluid mechanics, and quantum mechanics. Fractional calculus, as an extended form of integer calculus, can well characterize the memory characteristics, and fractal characteristics of the system [21,22,23]. Using the fractional calculus to model the heat conduction of the battery can more accurately describe the non-standard heat diffusion characteristics of the porous electrode of the battery. However, the materials of lithium-ion batteries are more complex, and the heat source of the battery changes in real time. Thus far, the fractional heat conduction model has not been applied to lithium-ion batteries.

In this paper, the fractional heat conduction model is used to study the heat transfer properties of batteries and improve the simulation accuracy of the temperature. Firstly, a fractional heat conduction model in one-dimensional space is established, and the method of solving the fractional differential equations is introduced. Then, the fractional heat conduction model of lithium-ion batteries is established according to the thermodynamics of the battery. The test platform of battery temperature was built, and the temperature variation of battery was collected. The thermophysical parameters of the battery and the fractional derivative order are brought into the model. Finally, the fractional heat conduction model was used to simulate the temperature of the battery. The fractional heat conduction model as also used to simulate the transient temperature field. The results show that the fractional heat conduction model can improve the accuracy of simulated temperature and can simulate the temperature field of the battery well. The fractional heat conduction model is of great significance to the study of heat transfer characteristics of batteries. Moreover, the application of the fractional heat conduction model in the battery can monitor the performance of the battery in real time and ensure the safety and reliability of the battery.

## 2. Fractional Heat Conduction Model

In this section, a one-dimensional heat conduction model is established based on the theory of fractional calculus. Then, the method of solving the fractional heat conduction model is introduced. Finally, the characteristics of the fractional heat conduction model are analyzed. The difference between the fractional heat conduction model and the integer-order heat conduction model is highlighted.

### 2.1. Numerical Solution of the Fractional Heat Conduction Equation

The fractional heat conduction model is an extension of the integer-order heat conduction model. Different from the integer-order Fourier heat transfer model, the fractional heat conduction model has a fractional derivative order. The mathematical expression of the fractional heat conduction model in one-dimensional space can be written as

(1) $$\begin{array}{cc} \frac{\partial T^{\alpha }\left(m,p\right)}{\partial p^{\alpha }} & ={\left(b\right)}^{2}\frac{\partial^{2}T\left(m,p\right)}{\partial m^{2}},0<m<L,p>0\\ T(0,p) & =T_{1},T\left(L,p\right)=T_{2},p>0\\ T(m,0) & =f(m),0⩽m⩽L \end{array}$$

where *T* is the temperature, *m* is the distance, *p* is the time, *b* is the thermal conductivity, and α∈R (0<α<2) is the fractional derivative order. When the fractional derivative order α is equal to 1, Formula (1) represents the Fourier heat conduction model. When the fractional derivative order α is equal to 2, Formula (1) represents the thermal wave model.

There are several methods to solve the fractional differential equations. In this paper, the Grünwald–Letnikov definition is used to solve the heat conduction equation [24,25]. The expression defined by Grünwald–Letnikov is as follows

(2) $$\begin{array}{c} \frac{\partial T^{\alpha }\left(m,p\right)}{\partial p^{\alpha }}=\sum_{j=1}^{N_{f}}bc_{j}T_{m,p-j} \end{array}$$

The value of $N_{f}$ shall be determined by the following relationship

(3) $$\begin{array}{c} N_{\left(f\right)}=min\left[\frac{p}{h},\frac{LM}{h}\right] \end{array}$$

where *h* is the time step and LM is the total simulation time. LM is also the memory length of the fractional heat conduction model.

The binomial coefficient $bc_{j}$ is calculated as follows

(4) $$\begin{array}{c} bc_{0}=1,bc_{j}=\left(1-\frac{1+\alpha }{j}\right)bc_{j-1},forj⩾1 \end{array}$$

The central difference method is used to solve the fractional differential equation. In the fractional heat conduction model, the temperature at time *p* and position *m* can be calculated by the following formula [26,27]

(5) $$\begin{array}{c} T_{m,p}=MT_{m-1,p-1}-\sum_{j=1}^{N_{f}}bc_{j}T_{m,p-j}-2MT_{m,p-1}+MT_{m+1,p-1} \end{array}$$

Module *M* is determined by the following relationship

(6) $$\begin{array}{c} M={\left(\frac{b}{\Delta m}\right)}^{2}\Delta p^{\alpha } \end{array}$$

where Δm is the distance step and Δp is the time step.

### 2.2. Characteristics of the Fractional Heat Conduction Model

**Figure 1:**
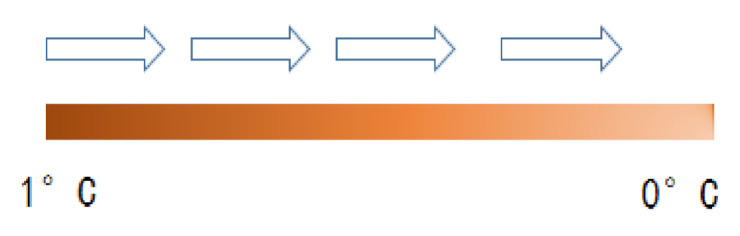
Fractional heat conduction in one-dimensional space.

The fractional heat conduction model can be used to analyze the heat conduction process in one-dimensional space. As shown in Figure 1, the left end of the rod has a constant temperature of 1 ${}^{\circ }$C, and the right end of the rod has a constant temperature of 0 ${}^{\circ }$C. The heat is conducted from the left end to the right end of the rod. The length of the rod is 2 m, and the rod is spatially divided into 20 sections at 0.1-m intervals. The fractional heat conduction equation of the rod is numerically solved. The whole simulation process lasts for 2 s, and the time step is 0.005 s. The memory length is 400, and the initial temperature is set at 0 ${}^{\circ }$C. The thermal conductivity in this section is selected according to the simulation time and time step. To facilitate the calculation, the simulation time is relatively short. If the selected thermal conductivity is not suitable, the difference between the fractional heat conduction model and the traditional heat conduction model can not be clearly distinguished. Therefore, to analyze the difference between the fractional heat conduction model and the traditional heat conduction model, the appropriate thermal conductivity is selected in this section. The thermal conductivity is set to 1 W/m·K.

**Figure 2:**
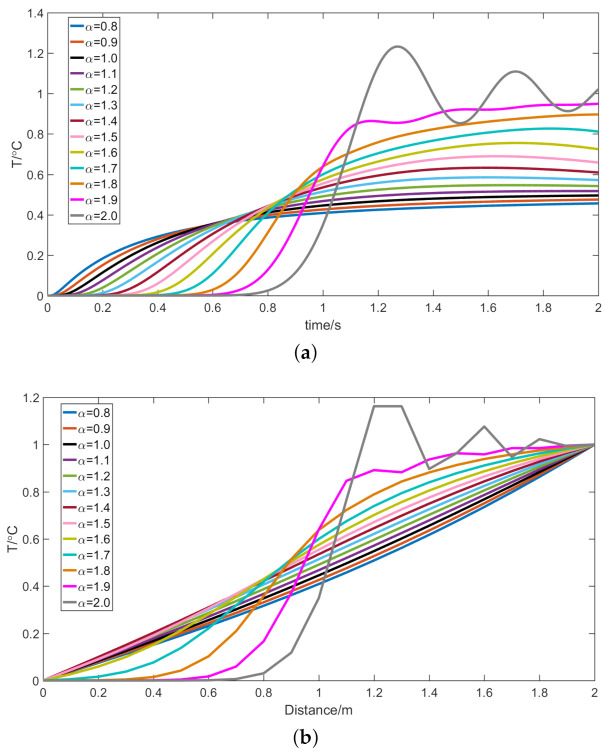
(**a**) Relationship between temperature and time under different fractional derivative orders (distance = 1 m); and (**b**) relationship between temperature and distance under different fractional derivative orders (time = 1 s).

Figure 2 shows the temperature variation under different fractional derivative orders. Figure 2a shows the temperature variation at the center of the rod (M = 1 m) and Figure 2b shows the temperature variation at 1 s. Figure 2 shows that, when the fractional derivative order is equal to 1, the fractional heat conduction model will be transformed into the Fourier heat transfer model. In the Fourier heat transfer model, the temperature rises rapidly at the initial moment and quickly reaches a steady state. At the initial moment of heat conduction, the rate of temperature rise will increase as the fractional derivative order of the fractional heat conduction model decreases. When the fractional derivative order is greater than 1, the form of heat transfer is changing. In addition, when the fractional derivative order of the fractional heat conduction model gradually increases, it takes longer for the temperature to reach a steady state. In the fractional heat conduction model, the temperature at certain moments will exceed the boundary value. When the fractional derivative order is equal to 2, the heat conduction model will be transformed into a thermal wave model. In the thermal wave model, the heat is transferred in the form of waves.

**Figure 3:**
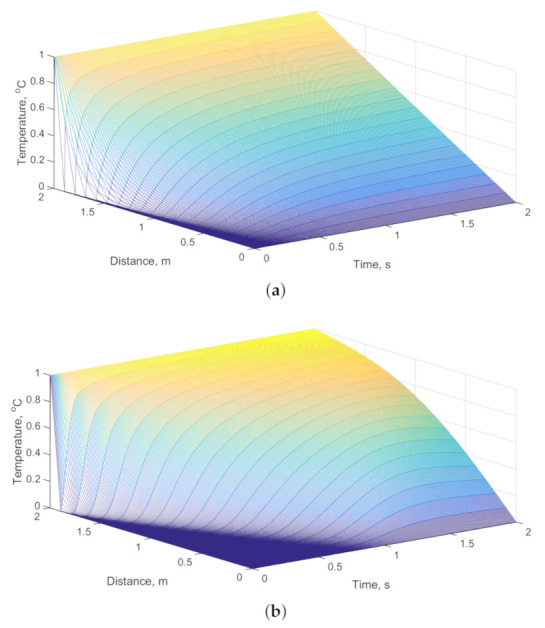

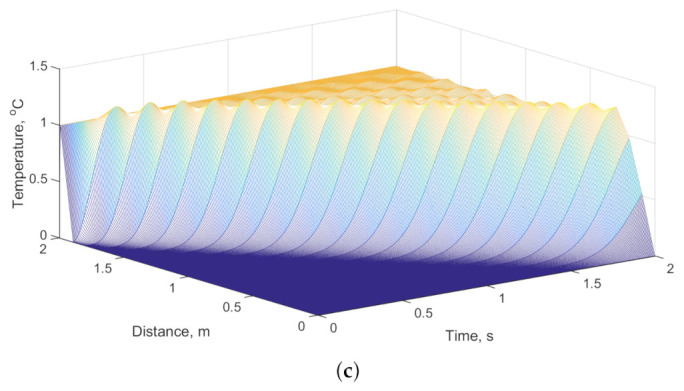
Temperature distribution of the rod in space and time: (**a**) fractional derivative order α = 1; (**b**) fractional derivative order α = 1.6; and (**c**) fractional derivative order α = 2.

Figure 3 shows the temperature variation at different spatial scales and temporal scales. Figure 3a–c shows the temperature variation when the fractional derivative order of the fractional heat conduction model is equal to 1, 1.6, and 2, respectively. The fractional derivative order in Figure 3a is equal to 1, which represents the Fourier heat conduction model. The Fourier thermal conductivity model is a standard diffusion model. Figure 3a shows that, at the last moment of the simulation, the temperature is proportional to the distance. The fractional derivative order of the fractional heat conduction model in Figure 3c is equal to 2, which represents the thermal wave model. Figure 3c shows that the heat is transferred in the form of waves. Compared with Fourier heat conduction model, the temperature in the thermal wave model takes longer to reach a steady state. At the initial moment, the rate of heat conduction in the thermal wave model is lower than that in the Fourier heat conduction model. In the thermal wave model, the heat has a significant hysteresis effect. Figure 3b represents the general form of the fractional heat conduction model. As the fractional derivative order of the fractional heat conduction model increases gradually, the low temperature region at the initial time increases gradually. The Fourier heat transfer model is a simple description of the heat transfer process. The thermal wave model is suitable for the ultra-high speed and ultra-low temperature environments, as well as for materials with poor thermal conductivity. The fractional heat transfer model is suitable for the microscopically discontinuous, porous, composite, and self-similar materials, and is a more accurate description of the heat transfer process. Fourier heat conduction model and the heat wave model are two extreme forms of the fractional heat conduction model. The fractional heat conduction model is an extended form of the traditional heat conduction models. By adjusting the fractional derivative order, the fractional heat conduction model can be more suitable for a variety of discontinuous materials.

## 3. Heat Conduction Model and Temperature Test of the Battery

In this section, a fractional heat conduction model for lithium-ion batteries is established. The thermophysical parameters of the battery are calculated. Then, the test platform was built to collect the data of the battery in the operation condition.

### 3.1. Fractional Heat Conduction Model of the Lithium-Ion Battery

Lithium-ion batteries are mainly composed of the electrodes, current collectors, separators, electrolyte, and casing. During the charging and discharging process, complex kinetic phenomena will occur inside the battery, including electrochemical and physical reactions. The heat transfer process of a battery is more complex than that of a single or composite material [28,29]. However, some of the dynamic phenomena in the battery have a small effect on the heat transfer and can be ignored. The heat conduction model of the battery needs to be simplified for the analysis and calculation of the heat transfer process. In this paper, it is assumed that the constituent materials of lithium-ion batteries are uniformly distributed. The density, specific heat capacity, and thermal conductivity of the battery are considered to be constant during the charging and discharging process. Whether the electrolyte is solid or liquid, the main heat transfer method inside the battery is heat conduction. The proportion of heat convection and heat radiation inside the battery is small, and it is not considered in most studies [30,31].

This paper assumes that heat is generated uniformly inside the battery and diffuses from the inside of the battery to the outside surface. The research object of this paper is the rectangular-shaped lithium-ion battery. The heat conduction formula of the three-dimensional transient differential form of the lithium-ion battery can be expressed as follows

(7) $$\begin{array}{cc} \rho c\frac{\partial^{\alpha }T}{\partial p^{\alpha }} & =\lambda_{x}\frac{\partial^{2}T}{\partial x^{2}}+\lambda_{y}\frac{\partial^{2}T}{\partial y^{2}}+\lambda_{z}\frac{\partial^{2}T}{\partial z^{2}}+q \end{array}$$

where ρ is the density; *c* is the average specific heat capacity at constant pressure; $\lambda_{x}$, $\lambda_{y}$, and $\lambda_{z}$ are the thermal conductivity of the battery in three coordinates; *T* is the temperature of the battery; *p* is time; and *q* is the heat generation rate of the battery.

The Grünwald–Letnikov definition is used to solve the fractional heat conduction model of the battery. The discrete form of Formula (7) can be written as follows

(8) $$\begin{array}{cc} \frac{T_{p,x,y,z}+\sum_{j=1}^{t/\Delta }bc_{j}T_{p-j,x,y,z}}{\Delta p^{\alpha }} & =\frac{\lambda_{x}}{\rho c}\times \frac{T_{p-1,x+1,y,z}-2T_{p-1,x,y,z}+T_{p-1,x-1,y,z}}{\Delta x^{2}}\\ \; & +\frac{\lambda_{y}}{\rho c}\times \frac{T_{p-1,x,y+1,z}-2T_{p-1,x,y,z}+T_{p-1,x,y-1,z}}{\Delta y^{2}}\\ \; & +\frac{\lambda_{z}}{\rho c}\times \frac{T_{p-1,x,y,z+1}-2T_{p-1,x,y,z}+T_{p-1,x,y,z-1}}{\Delta z^{2}}+q \end{array}$$

where Δp is the time step and Δx, Δy, and Δz are the steps in the *x*, *y*, and *z* directions, respectively.

The boundary condition of the thermal conduction of lithium-ion batteries satisfies Newton’s law of cooling, which can be written as follows [31,32,33]

(9) $$\begin{array}{cc} \; & -\lambda_{x}\frac{\partial^{2}T}{\partial x^{2}}=h_{c}\left(T-T_{\infty }\right),x=0orl_{b}\\ \; & -\lambda_{y}\frac{\partial^{2}T}{\partial y^{2}}=h_{c}\left(T-T_{\infty }\right),y=0orb_{b}\\ \; & -\lambda_{z}\frac{\partial^{2}T}{\partial z^{2}}=h_{c}\left(T-T_{\infty }\right),z=0orh_{b} \end{array}$$

where $h_{c}$ is the heat transfer coefficient; $T_{\infty }$ is the ambient temperature; and $l_{b}$, $b_{b}$, and $h_{b}$ are the length, width and height of the battery, respectively.

### 3.2. Thermal Properties Parameters of the Lithium-Ion Battery

The most commonly used method for calculating the thermal conductivity of lithium-ion batteries is the equivalent resistance method. This paper assumes that the plates of the battery are perpendicular to the *z*-axis [31,32,33]. The equivalent calculation method of thermal conductivity can be expressed as:

(10) $$\begin{array}{cc} \; & \lambda_{x}=\sum_{}^{i}\frac{\lambda_{i}d_{x_{i}}}{l_{b}}=\frac{\lambda_{p}d_{x_{p}}+\lambda_{n}d_{x_{n}}+\lambda_{s}d_{x_{s}}}{l_{b}}\\ \; & \lambda_{y}=\sum_{}^{i}\frac{\lambda_{i}d_{y_{i}}}{b_{b}}=\frac{\lambda_{p}d_{y_{p}}+\lambda_{n}d_{y_{n}}+\lambda_{s}d_{y_{s}}}{b_{b}}\\ \; & \lambda_{z}=\frac{h_{b}}{\sum_{}^{i}\frac{d_{z_{i}}}{\lambda_{i}}}=\frac{h_{b}}{\frac{d_{z_{p}}}{\lambda_{p}}+\frac{d_{z_{n}}}{\lambda_{n}}+\frac{d_{z_{s}}}{\lambda_{s}}} \end{array}$$

where $\lambda_{p}$, $\lambda_{n}$, and $\lambda_{s}$ are the thermal conductivity of the positive electrode plate, negative electrode plate, and separator in the *z*-axis direction, respectively; $d_{x_{p}}$, $d_{x_{n}}$, and $d_{x_{s}}$ are the thickness of the positive electrode plate, negative electrode plate, and separator in the x-axis direction, respectively; and $d_{y_{i}}$ and $d_{z_{i}}$ are the thickness in the y-axis and *z*-axis directions, respectively.

The specific heat capacity is one of the important thermophysical parameters of the battery. In this paper, the specific heat capacity of each component of the battery is measured, and then the specific heat capacity is obtained by the following formula

(11) $$\begin{array}{c} c_{p}=\frac{1}{m}\sum_{}^{i}c_{i}m_{i} \end{array}$$

where $c_{i}$ represents the specific heat capacity of each component of the battery, $m_{i}$ represents the mass of each component of the battery, and *m* represents the mass of the battery.

The calculation formula for the equivalent density of the battery is as follows

(12) $$\begin{array}{c} \rho =\frac{\sum_{}^{i}m_{i}}{\sum_{}^{i}v_{i}} \end{array}$$

where $m_{i}$ represents the mass of each component of the battery and $v_{i}$ represents the volume of each component of the battery.

It is generally believed that the heat of a battery mainly includes the enthalpy heat, Joule heat, polarization heat, and reaction heat. These four parts of heat are caused by the reversible reactions, internal resistance, polarization resistance, and side reactions. Currently, the Bernardi heat generation model is often used to estimate the heat generation rate of the battery [31,32,33]. The heat generation rate *q* of the battery can be expressed as follows

(13) $$\begin{array}{c} q=\frac{1}{V_{b}}\left[I^{2}R_{total}-IT\frac{dE_{0}}{dT}\right] \end{array}$$

where *I* is the current of the battery, $R_{total}$ is the internal resistance of the battery, $V_{b}$ is the volume of the battery, and $dE_{0}/dT$ is the influence coefficient of temperature.

**Figure 4:**
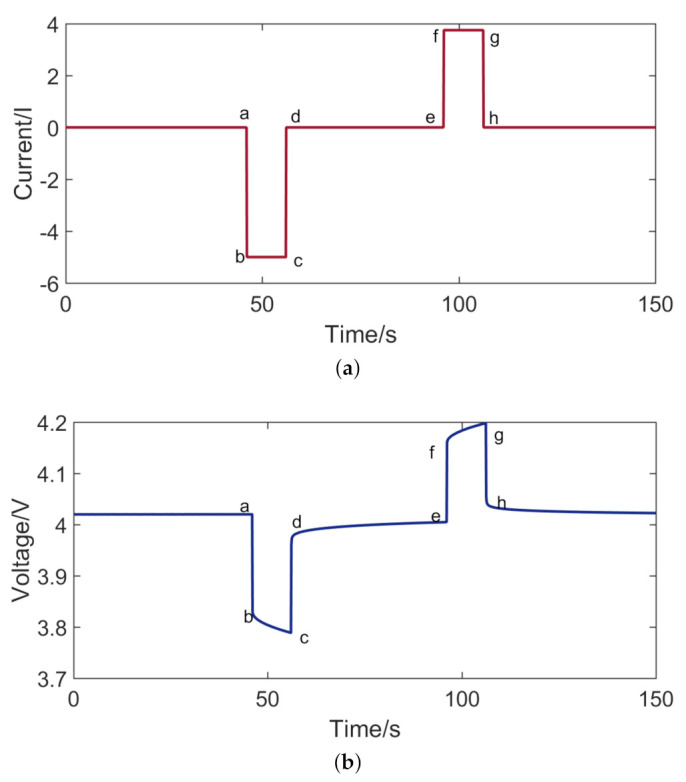
Hybrid pulse power characteristic test: (**a**) current; and (**b**) voltage.

The common method for calculating the internal resistance of the battery is the hybrid pulse power characteristic test. The current and voltage of the hybrid pulse power characteristic test are shown in Figure 4. When a pulse current is applied to the battery, the voltage will have a mutation, as shown in the voltage variation between Point a and Point b. The voltage variation between Point a and Point b is caused by the ohmic resistance of the battery. The voltage variation between Point b and Point c is caused by the polarization resistance of the battery.

The internal resistance of a lithium ion battery is calculated as follows

(14) $$\begin{array}{cc} R_{dis} & =\frac{U_{c}-U_{a}}{I_{ab}}\\ R_{cha} & =\frac{U_{g}-U_{e}}{I_{fg}}\\ R_{total} & =\frac{R_{dis}+R_{cha}}{2}\times 100\% \end{array}$$

where $R_{dis}$ represents the internal resistance during the discharge process and $R_{cha}$ represents the internal resistance during the charge process.

**Figure 5:**
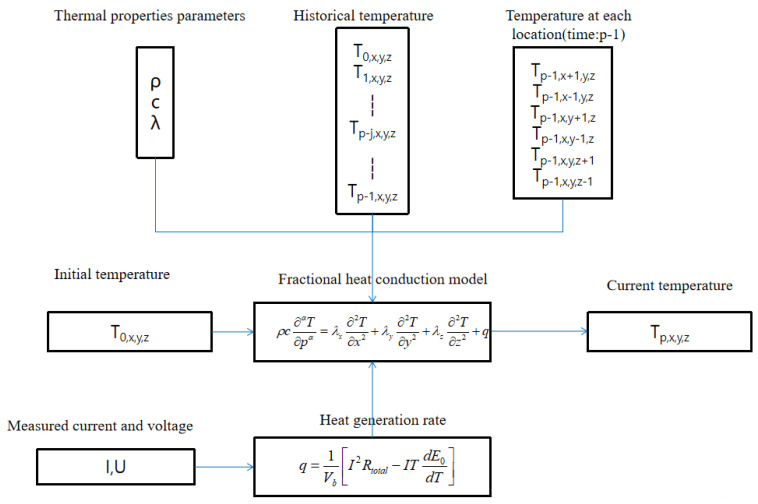
The block diagram of the numerical algorithm of the fractional heat conduction model.

The block diagram of the numerical algorithm of the fractional heat conduction model is shown Figure 5.

### 3.3. Temperature Characteristic Test of the Lithium-Ion Battery

**Figure 6:**
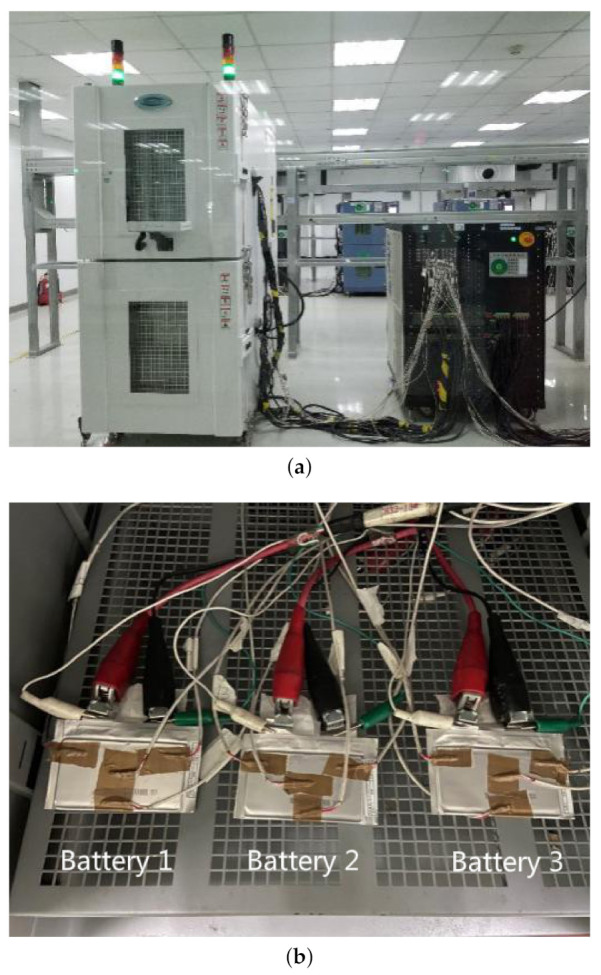
(**a**) Experimental platform of lithium-ion batteries; and (**b**) the lithium-ion batteries to be tested.

In the experiment, a computer-controlled test platform (Arbin BT2000 tester) was used to charge and discharge the lithium-ion batteries. The experimental platform of lithium-ion battery is shown in Figure 6. The test platform simultaneously records the current and voltage of the battery. The test platform adopts the corresponding software to control the engineering department of the experiment and save the measured data. The collected data include the current, voltage, and temperature. The lithium-ion battery to be tested is placed in a thermostat. The thermocouples are attached to the surface of the battery to collect the temperature at various locations. The sampling frequency in the experiment was 1 HZ. The temperature sensor is shown in Figure 6b.

**Figure 7:**
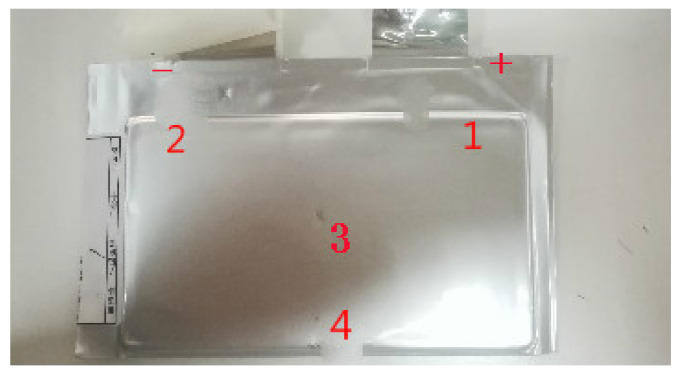
Temperature acquisition points of the battery.

Three lithium-ion batteries (LiNi${}_{x}$Mn${}_{y}$Co${}_{z}$O${}_{2}$) were used for testing. The three batteries were labeled as Batteries 1–3. The electrolyte is mainly composed of the lithium salt and organic solvent. Figure 7 shows the temperature acquisition points of the battery. Each temperature acquisition point is equipped with a temperature sensor. The test platform uses the platinum temperature sensors and paperless temperature recorders. The accuracy of the temperature sensor is 0.15 + 0.002 × |T|. The lithium-ion battery is placed vertically on the porous frame of the temperature chamber. The batteries are preheated before the formal test. The temperature acquisition system includes a platinum temperature sensor and a computer [34].

Table 1 lists the basic parameters of the tested battery. The thermophysical parameters of each component of the lithium-ion battery are shown in Table 2. The thermal physical parameters of lithium-ion batteries are shown in Table 3. The parameters in Table 2 were set according to the battery manufacturer and those found in [35,36,37,38,39,40,41]. The thermophysical parameters in Table 3 were calculated according to Formulas (10)–(12).

The experiments in this paper include the charge and discharge tests at different temperatures, the charge and discharge tests at different discharge rates, and the hybrid pulse power characteristic test. The charge and discharge tests at different temperatures and different rates were used to verify the battery model and analyze the simulation effect of the battery temperature field. After the temperature of the incubator reached a stable state, the charge and discharge test was carried out. The constant current charge and discharge tests were carried out at different ambient temperatures. The specific steps are as follows

- The thermostat was set to a constant temperature (*T*). The battery was charged to the upper voltage limit by a constant current (1 C rate). Then, the battery was continuously charged until the current was less than 0.05 C (constant current and constant voltage mode). Finally, the load current of the battery was cut off and the battery was allowed to stand for a certain period of time (1 h).
- The battery was discharged to the cut-off voltage by a current of 1 C.
- The thermostat was set to different ambient temperatures (*T* = 0, 25, and 40 ${}^{\circ }$C). The battery was preheated in the incubator for 5 h. Then, the above steps were repeated.

**Figure 8:**
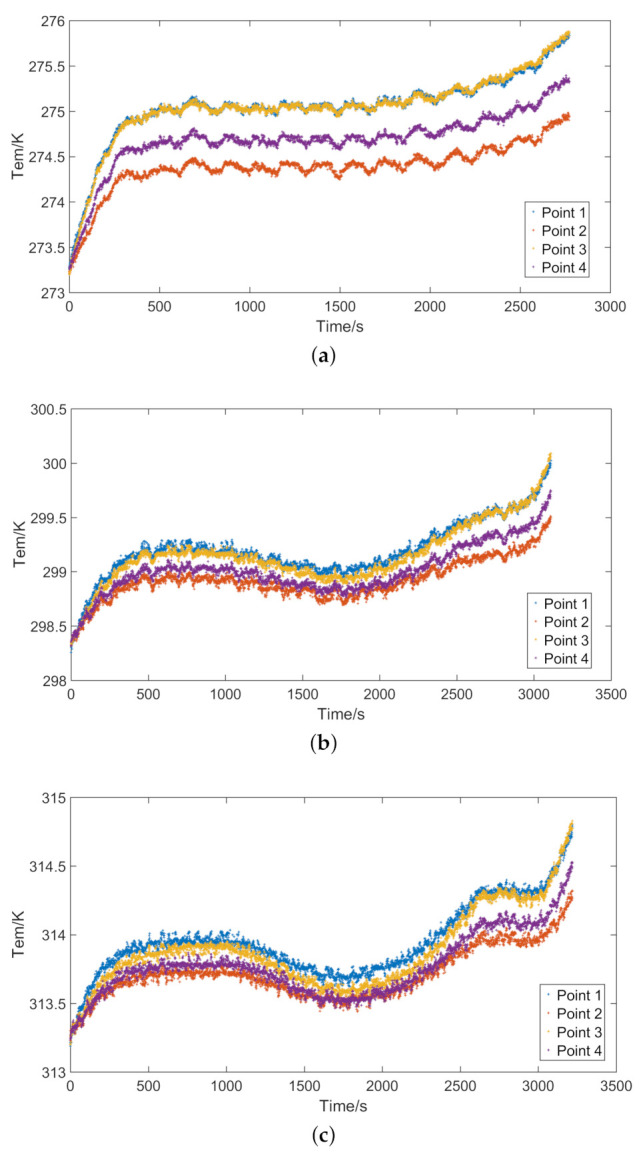
Temperature of the battery at different ambient temperatures: (**a**) ambient temperature *T* = 0 ${}^{\circ }$C; (**b**) ambient temperature *T* = 25 ${}^{\circ }$C; and (**c**) ambient temperature *T* = 40 ${}^{\circ }$C.

When the ambient temperature of the tested lithium-ion battery is set at 0, 25, and 40 ${}^{\circ }$C, the corresponding capacities are 3.99, 4.39, and 4.52 Ah, respectively. The discharge capacity of the lithium-ion battery in low-temperature environment will be reduced, which is mainly due to the decrease of electrochemical reaction rate. The temperature of the battery at different ambient temperatures is shown in Figure 8. Figure 8 shows that the discharge time of the battery is the shortest at 0 ${}^{\circ }$C. Compared with 25 and 40 ${}^{\circ }$C, the battery reaches the cut-off voltage earlier at 0 ${}^{\circ }$C. The temperature of the battery is closely related to the internal resistance. In the initial stage of the discharge, the internal resistance of the battery will increase, and the battery temperature will also gradually rise. In the middle stage of the discharge, the internal resistance of the battery changes little, so the battery temperature remains in a stable state. At a higher ambient temperature, the temperature of the battery in the middle of the discharge will drop slightly, which is caused by the polarization effect inside the battery. At the end of discharge, the internal resistance of the battery rises again, causing the temperature to rise. The temperature of the entire discharge process is recorded on the test platform. Because the sampling frequency is high and many data are collected, the test platform can accurately record the temperature changes of the battery. Before the temperature simulation calculation, the data were optimized. After the battery is fully preheated, the temperature difference among the surface of the battery, the temperature sensor, and the battery tester is very small. This paper does not consider the influence of battery tester on the heat conduction of battery.

It is worth noting that the temperature at Point 1 is higher than the temperature at other test points. Because the heat generated by the positive electrode of the battery affects the temperature at Point 1, the temperature at Point 1 rises slightly. Therefore, the heat generated by the electrodes of the battery needs to be loaded to the test point. The heat generated by the electrode can be calculated by the following formula

(15) $$\begin{array}{c} q_{ele}=\frac{I^{2}R_{ele}}{V_{ele}} \end{array}$$

where $q_{ele}$ is the heat generation rate of the electrodes, $R_{ele}$ is the resistance of the electrodes, and $V_{ele}$ is the volume of the electrodes.

The heat generation rate of a lithium-ion battery is directly related to the charge and discharge current of the battery. Therefore, it is necessary to measure the temperature variation of the battery at different discharge rates. The test steps of the battery under different discharge rates are as follows:

- The thermostat was set to a constant temperature (25 ${}^{\circ }$C). The battery was charged to the upper voltage limit by a constant current (0.5 C rate). Then, the battery was continuously charged until the current is less than 0.05 C (constant current and constant voltage mode). Finally, the load current of the battery was cut off and the battery was allowed to stand for a certain period of time(1 h).
- The battery was discharged to the cut-off voltage by a current of 2 C.
- The discharge rate of the battery is set to 3 C. Then, the above steps were repeated.

**Figure 9:**
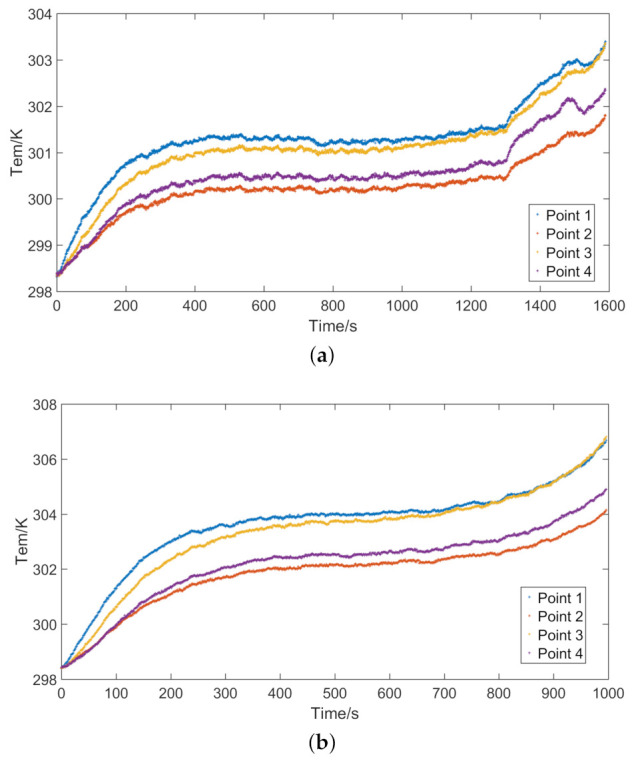
Temperature of the battery at different discharge rates: (**a**) current *I* = 2 C; and (**b**) current *I* = 3 C.

The temperature of the battery at different discharge rates is shown in Figure 9. The discharge time of the battery will decrease as the discharge rate increases.

The steps of the hybrid pulse power characteristic test are as follows:

1. The thermostat was set to a constant temperature (*T*). The battery was charged to the upper voltage limit by a constant current (1 C rate). Then, the battery was continuously charged until the current is less than 0.05 C (constant current and constant voltage mode). Finally, the load current of the battery was cut off and the battery was allowed to stand for a certain period of time (1 h).
2. The battery was discharged by 1C rate current until the state of charge drops by 10%. The battery was then allowed to stand for 1 h.
3. The battery was discharged by 1C rate current for 10 s, and then the battery was allowed to stand for 40 s. The battery was charged by 0.75 C current for 10 s and then allowed to stand for 1 h.
4. Steps 2 and 3 were repeated until the voltage reached the cut-off voltage of the battery.
5. The thermostat was set to different ambient temperatures (*T* = 0, 25, and 40 ${}^{\circ }$C). The battery as preheated in the incubator for 5 h. Then, the above steps were repeated.

**Figure 10:**
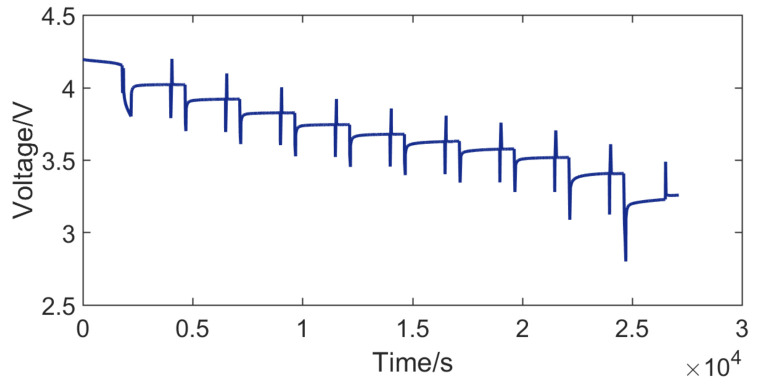
Voltage of the hybrid pulse power characteristic test (ambient temperature *T* = 0 ${}^{\circ }$C).

**Figure 11:**
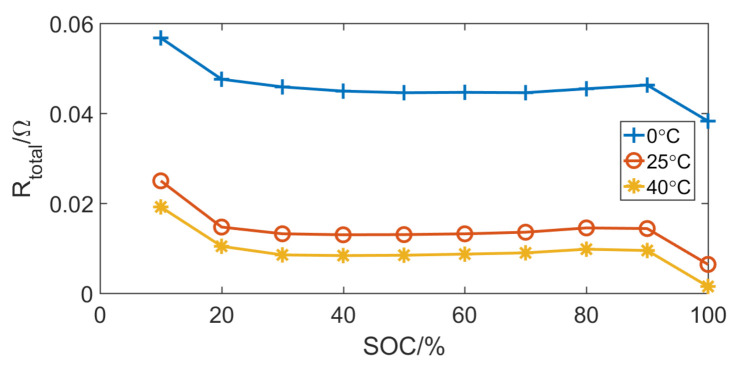
Internal resistance of the battery at different ambient temperatures.

Figure 10 shows the voltage of the hybrid pulse power characteristic test. According to Formula (14), the internal resistance of the battery can be calculated. Figure 11 shows the internal resistance of the battery at different ambient temperatures.

The state of charge (SOC) of the battery is defined as the percentage of the remaining capacity in the total capacity, which is expressed as

(16) $$\begin{array}{c} SOC=(1-\frac{It}{3600C_{total}})\times 100\% \end{array}$$

where $C_{total}$ is the total rated capacity of the battery.

The relationship between the internal resistance of the battery and the SOC can be fitted with a polynomial, and the expression is as follows

(17) $$\begin{array}{c} R_{total}=A_{0}+A_{1}SOC+A_{2}SOC^{2}+A_{3}SOC^{4} \end{array}$$

where $A_{0}$, $A_{1}$, $A_{2}$, and $A_{3}$ are the polynomial coefficients.

According to Formulas (14)–(17), the heat generation rate of the battery can be calculated.

## 4. Simulation of the Temperature Field of Lithium-Ion Batteries

In this section, the thermophysical parameters and heat source of the battery are brought into the fractional heat conduction model for calculation. The temperature at different test points of the battery is used to verify the accuracy of the fractional heat conduction model. In addition, the fractional heat conduction model is used to simulate the transient temperature field of the battery.

### 4.1. Temperature Simulation

**Figure 12:**
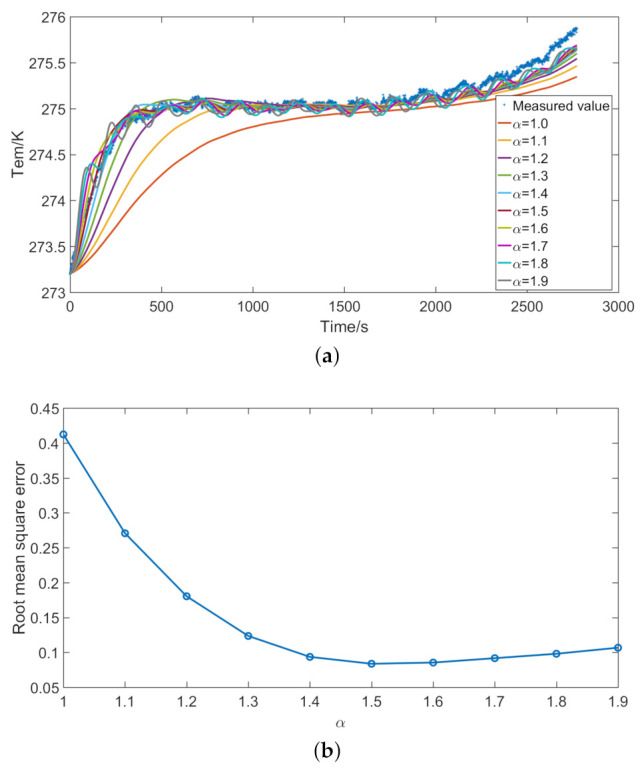
Simulation results of the battery at an ambient temperature of 0 ${}^{\circ }$C: (**a**) temperature; and (**b**) root mean square error.

According to Formula (8), the difference method is used to establish the heat conduction model in the numerical analysis software (Matlab). The length of the battery is divided into 25 parts with a step length of 0.005 m. The width of the battery is divided into 17 parts with a step length of 0.005 m. The height direction of the battery is divided into three parts with a step length of 0.002 m. The total simulation time is consistent with the discharge time, and the time step is 1 s. The memory length of the fractional heat conduction model is the total simulation time. The thermophysical parameters in the model are set according to Table 3. During the experiment, the tape was used at the temperature collection point. The heat transfer coefficient between the battery and the tape is brought into the boundary condition. The initial temperature is the surface temperature of the battery. The fractional derivative order is brought into the model for calculation, and the simulated temperature can be obtained. Figure 12 shows the simulation results of the battery at an ambient temperature of 0 ${}^{\circ }$C.

Figure 12a shows the simulated temperature at different fractional derivative orders. Figure 12a shows that the fractional derivative order of heat conduction model has a great influence on the simulated temperature. The traditional heat conduction model in this paper refers to Fourier heat conduction model. Fractional heat conduction model is an extension of Fourier heat conduction model. When the fractional derivative order is equal to 1, the fractional heat conduction model represents the Fourier heat conduction model. When the fractional derivative order is equal to 1, the error between the simulated temperature and the measured temperature is large. Only when the discharge time is more than 1000 s can the Fourier heat conduction model simulate the measured temperature well. The Fourier heat conduction model is a standard form of diffusion, which is difficult to apply to heat conduction in porous materials. As the fractional derivative order increases, the error between the measured temperature and the simulated temperature gradually decreases. Figure 12b shows the root mean square error between the measured and simulated temperatures.

When the fractional derivative order is equal to 1.5, the error between simulated temperature and measured temperature is very small. The fractional heat conduction model can approximate the measured temperature in a very short time. When the fractional derivative order is between 1.5 and 1.9, the root mean square error between the measured temperature and the simulated temperature varies little. In addition, when the fractional derivative order is greater than 1.5, the simulated temperature fluctuates obviously. The fluctuation of temperature is caused by several factors, such as the change of internal resistance, the polarization effect, and the porous electrode material. Moreover, the materials of the positive electrode, negative electrode, and separator of the battery are different. Compared with the Fourier heat transfer model, the fractional heat conduction model can better simulate the complex thermodynamic phenomena inside the battery. The fractional derivative order is related to the material and structure of the battery. When the fractional derivative order is equal to 1.5, it is more suitable for the heat conduction model of the battery in this paper. The optimal fractional derivative order of the lithium-ion battery in this paper is 1.5. The optimal fractional derivative order varies with the type of battery. The optimal fractional derivative order 1.5 is only applicable to the lithium-ion batteries tested in this paper. Therefore, the accuracy of fractional heat conduction model (fractional derivative order α≠ 1) is higher than that of Fourier heat conduction model (fractional derivative order α = 1). Therefore, the fractional derivative order of the battery model is set to 1.5.

**Figure 13:**
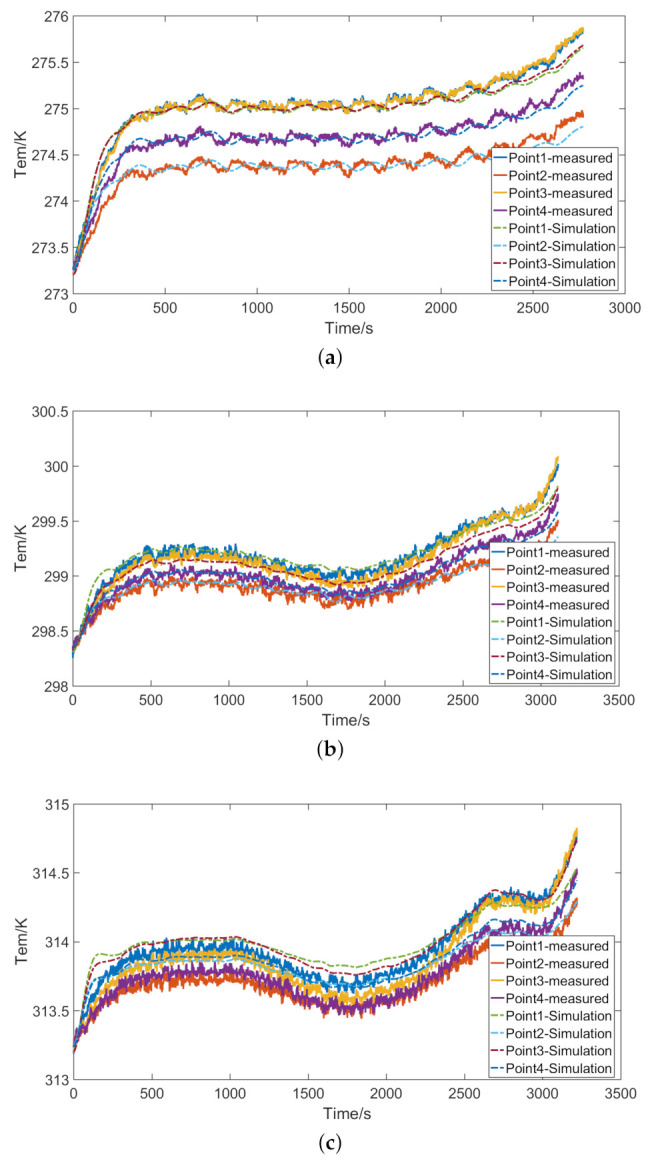
Simulated temperature at different ambient temperatures: (**a**) ambient temperature *T* = 0 ${}^{\circ }$C; (**b**) ambient temperature *T* = 25 ${}^{\circ }$C; and (**c**) ambient temperature *T* = 40 ${}^{\circ }$C.

**Figure 14:**
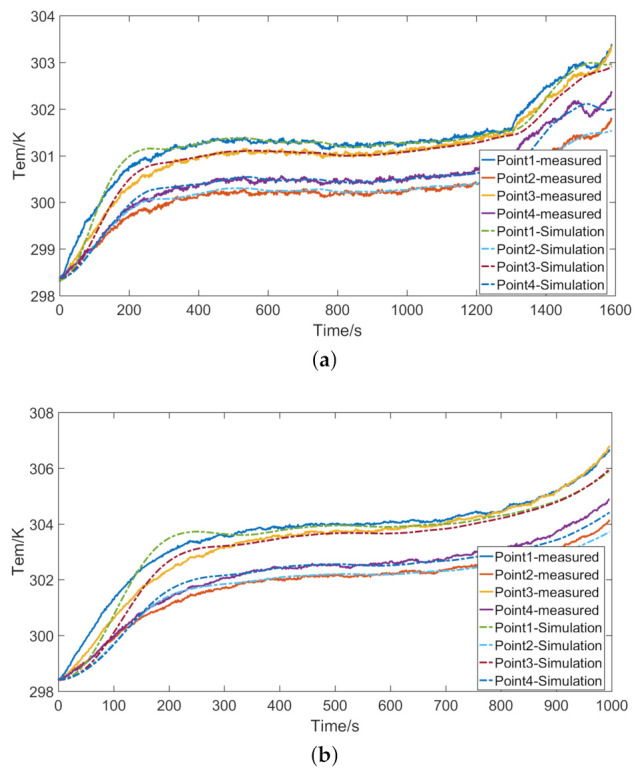
Simulated temperature at different discharge rates: (**a**) current *I* = 2 C; and (**b**) current *I* = 3 C.

Figure 13 shows the simulated temperature at different ambient temperatures. Figure 14 shows the simulated temperature at different discharge rates. In the initial stage of discharge, there is an obvious error between the simulated temperature and the measured temperature. In the middle of the discharge, the simulated temperature is relatively stable and has high accuracy because the internal resistance of the battery at this time has stabilized.

### 4.2. Transient Temperature Field of the Battery

**Figure 15:**
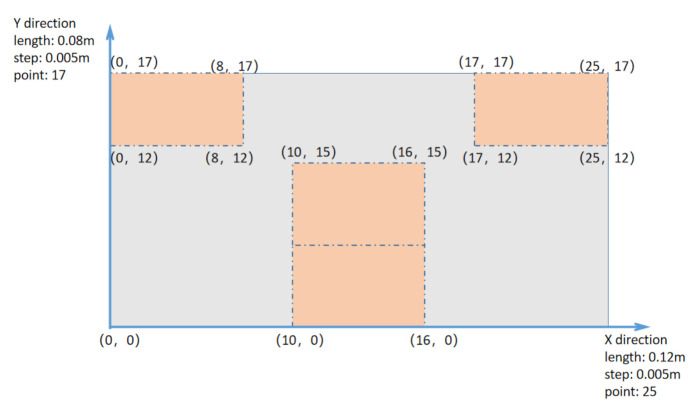
Location of the tape on the surface of the battery.

The transient temperature field of the battery can clearly understand the temperature distribution of the battery. During the experiment, some areas of the battery surface are not in direct contact with the air. The temperature distribution is related to the size of the tape and the location of the tape. In the area covered by the tape, the heat dissipation of the battery will be poor. The temperature of the area covered by the tape will be slightly higher than the temperature of other areas. The heat exchange coefficient of the tape covered area is different from those of other areas. The influence of the tape on the heat dissipation of the battery should be considered. Figure 15 shows the location of the tape on the surface of Battery 1. The red area represents the area covered by tape, while the gray area represents the area covered by air. The heat transfer coefficient is selected according to the empirical value. The heat transfer coefficient ($h_{c}$) between the battery and the air is set to 5 W·m${}^{-2}$·K${}^{-1}$. In the gray area, the heat transfer coefficient ($h_{c}$) is brought into the cooling condition. In the red area, a new heat transfer coefficient is brought into the cooling condition. The new heat transfer coefficient takes into account the effect of the tape on the heat dissipation of the battery. The new heat transfer coefficient is set to 0.72$h_{c}$.

**Figure 16:**
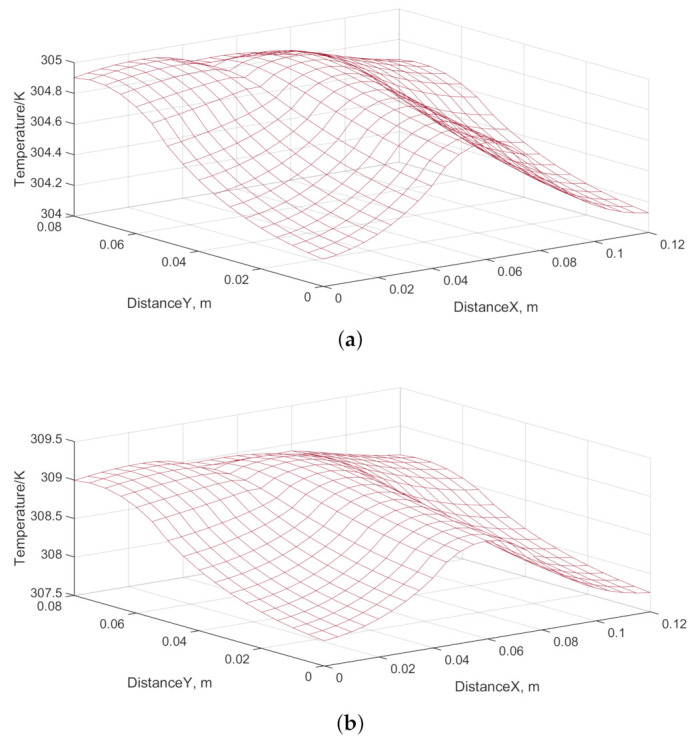
Simulated Temperature field of the battery: (**a**) current *I* = 2 C; and (**b**) current *I* = 3 C.

**Figure 17:**
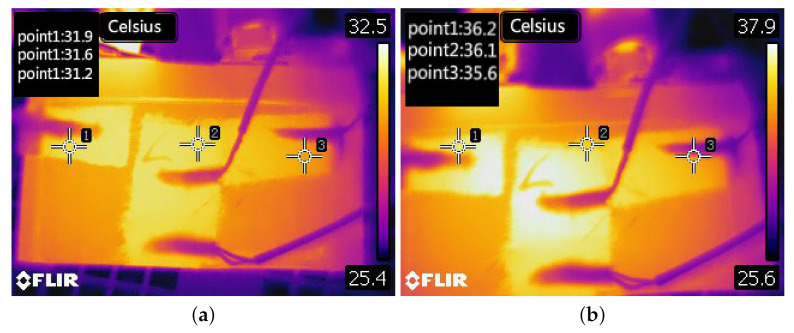
Thermal image of the battery: (**a**) current *I* = 2 C; and (**b**) current *I* = 3 C.

The temperature field of the battery at the end of the simulation is shown in Figure 16. The thermal image of the battery is shown in Figure 17. Figure 17 shows the temperature distribution of Battery 1. The distribution of the tape of Battery 1 presents the T-shape. Figure 17 shows that the simulated temperature field is consistent with the measured temperature distribution of the battery. The increased temperature in Figure 17 also shows the T-shape.

**Figure 18:**
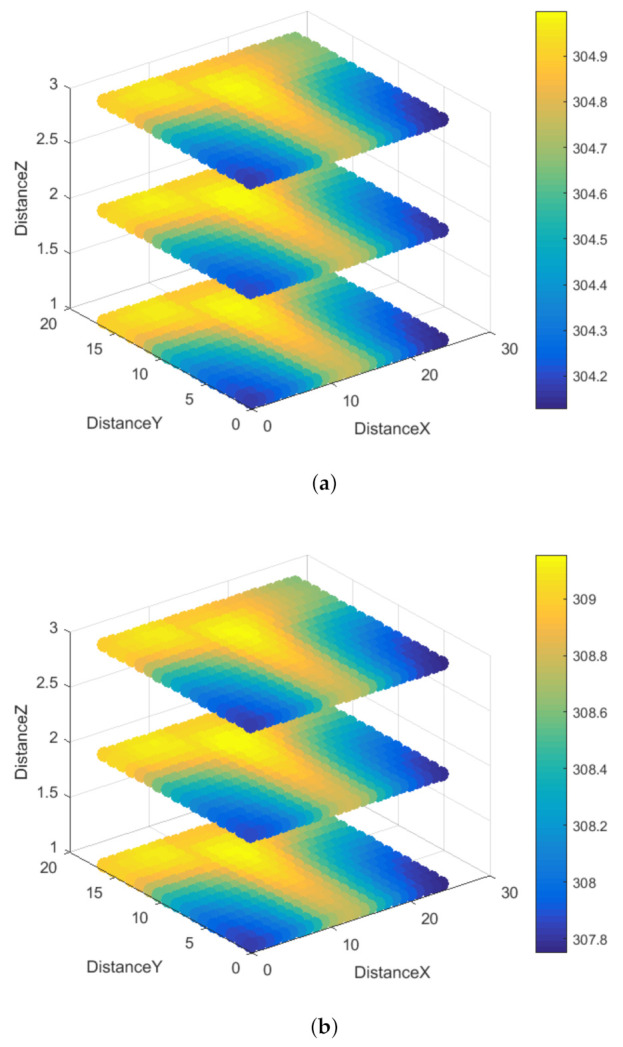
Scatter plot of the simulated temperature: (**a**) current *I* = 2 C; and (**b**) current *I* = 3 C.

Figure 18 shows a scatter plot of the simulated temperature. Figure 18 shows that the temperature distribution of the battery is basically consistent with the simulation results.

## 5. Conclusions

The electrodes of lithium-ion batteries are made of porous materials. The heat transfer in lithium-ion batteries has nonlinear characteristics. The traditional integer-order heat conduction model is not suitable for describing the thermal dynamics of the battery. In this paper, a fractional heat conduction model is used to model the lithium-ion battery. The simulation results of the battery temperature show that the fractional heat conduction model has high accuracy. Compared with the integer-order heat conduction model, the fractional-order heat conduction model can approximate the measured temperature more quickly. The fractional heat conduction model is of great significance to the study of the temperature distribution of the battery. The experiment presented in this paper could not fully simulate the actual working conditions of electric vehicle battery. In the future research, we will optimize the experimental design and analyze the characteristics of fractional heat conduction in the actual working environment.

## Figures and Tables

**Table 1:** Specification of the lithium-ion battery.

Battery	Nominal Capacity (Ah)	Operating Voltage (V)	Length, Width and Height (mm)	Weight (g)	Volume (cm${}^{3}$)
LiNi${}_{x}$Mn${}_{y}$Co${}_{z}$O${}_{2}$	5	2.8–4.2	125, 86, and 5.6	82	36.3

**Table 2:** Thermal physical parameters of each component of the lithium-ion battery.

Composition	Material	Specific Heat Capacity (J/(kg·K))	Thermal Conductivity (W/m·K)	Density (kg/cm${}^{3}$)	Thickness (um)
Shell	Aluminum plastic film	1376.947	0.427	1636.0	145
Separator	Polypropylene	1978.16	0.3344	648.773	40
Cathode	Ternary materials	1067.6	2.7	2584.25	40
Anode	Graphite	1064.0	1671.24	3.3	35
Electrode conductor (+)	Aluminum	903.0	238.0	2702.0	-
Electrode conductor (−)	Copper	385.0	398.0	8933.0	-

**Table 3:** Thermal physical parameters of lithium-ion batteries.

Equivalent Specific Heat Capacity (J/(kg·K))	Equivalent Thermal Conductivity (W/m·K)	Equivalent Density (kg/cm${}^{3}$)	Temperature Influence Coefficient (mV·/K)
944.24	27.242 (x,y) − 3.583 (z)	2.1339	0.279

## Data Availability

Restrictions apply to the availability of these data. Data was obtained from SVOLT Energy Technology Co., Ltd., Wuxi, China.
